# Brief Exercise at Work (BE@Work): A Mixed-Methods Pilot Trial of a Workplace High-Intensity Interval Training Intervention

**DOI:** 10.3389/fspor.2021.699608

**Published:** 2021-07-02

**Authors:** Naomi L. Burn, Matthew Weston, Greg Atkinson, Michael Graham, Kathryn L. Weston

**Affiliations:** ^1^School of Health and Life Sciences, Teesside University, Middlesbrough, United Kingdom; ^2^School of Applied Sciences, Edinburgh Napier University, Edinburgh, United Kingdom

**Keywords:** high-intensity interval training, workplace, pilot trial, mixed method, intervention

## Abstract

**Introduction:** The efficacy of high-intensity interval training (HIIT) for improving markers of physical fitness and cardiometabolic health is promising. The workplace is one non-laboratory setting where the effectiveness of HIIT could be explored. The aim of this study was to undertake a mixed-methods exploratory pilot trial of a workplace HIIT intervention named Brief Exercise at Work (BE@Work).

**Methods:** Fifty-four healthy employees (mean ± standard deviation [SD] age 46 ± 10 years) from two workplaces in Northeast England were allocated to 8 weeks of thrice-weekly workplace HIIT based on boxing, stair climbing and stepping, comprising 4–7 60 s high-intensity intervals interspersed with 75 s rest (*n* = 30), or a no-intervention control (*n* = 24). The primary outcome was the change SD of predicted maximal oxygen consumption (VO_2max_). Markers of physical fitness, cardiometabolic health and mental well-being were also measured at baseline and follow-up. Participant perceptions of the intervention were explored in post-intervention focus groups (*n* = 9).

**Results:** Mean (±SD) session attendance was 82% (±15%). Mean peak heart rate across the intervention was 87% of age-predicted maximal heart rate with a within- and between-subject SD of 5.5% and 3.7%, respectively. The SD of changes in predicted VO_2max_ was 6.6 mL·kg^−1^·min^−1^ across both groups, which can be used to inform sample size estimations for a future full trial. The control-adjusted mean increase (95% confidence interval) in predicted VO_2max_ was 3.9 (−0.2 to 8.1) mL·kg^−1^·min^−1^, corresponding to a Cohen's D of 0.47. We also observed preliminary evidence of small to moderate effects in favour of the intervention group for non-dominant leg extensor muscle power, markers of health-related quality of life, well-being and perceived stress and small to moderate effects in favour of the controls in perceived pain, physical activity and high-density lipoprotein cholesterol. During HIIT, focus group participants reported physiological responses they perceived as unpleasant or tiring (e.g., breathlessness, local muscular fatigue), but also that they felt alert and energised afterwards.

**Conclusion:** The findings of this exploratory pilot trial support the implementation of a definitive randomised controlled trial to quantify the effectiveness of a workplace HIIT intervention.

## Introduction

High-intensity interval training (HIIT) is a form of exercise characterised by brief, intermittent bouts of intense exercise, usually at ≥85% maximal heart rate (HR_max_) (Weston K. S. et al., 2014), alternated with periods of rest or low intensity recovery (Gibala et al., 2012). The positive effects of HIIT on cardiorespiratory fitness (Weston, M. et al., 2014) and markers of cardiometabolic health (Su et al., 2019) in laboratory environments are promising. Furthermore, adaptations are typically seen with a lower total exercise time commitment than continuous exercise at lower relative intensities (Milanovic et al., 2015). Despite this, the effectiveness and feasibility of HIIT outside the laboratory has been questioned (Biddle and Batterham, 2015). Opponents of HIIT do not contest its utility for eliciting improvements in health and fitness variables in tightly controlled laboratory environments (Biddle and Batterham, 2015) [i.e., HIIT efficacy (Courneya, 2010)], yet there is dispute surrounding the potential effectiveness of HIIT in “real-world” settings with minimal supervision and exercise equipment (Biddle and Batterham, 2015).

The workplace is one “real-world” setting that provides a relatively controlled environment for the implementation of health promotion initiatives (National Institute for Health Care Excellence, 2008). Indeed, systematic reviews demonstrate that exercise delivered in the workplace can improve cardiorespiratory fitness (Burn et al., 2019), cardiometabolic health (Reed et al., 2017) and well-being (Abdin et al., 2018) but the weekly time commitment typically required for workplace exercise interventions is ~80 min (Burn et al., 2019). Given that lack of time has been reported as a barrier to workplace exercise participation (Hunter et al., 2018), more time efficient exercise strategies such as HIIT could be well-received by organisations and employees alike. The effectiveness of workplace HIIT has begun to be explored in a small number of pilot trials (Shepherd et al., 2015; Allison et al., 2017; Cuddy et al., 2019; Eather et al., 2020; Metcalfe et al., 2020). This preliminary body of work suggests that workplace HIIT may elicit improvements in physical fitness and cardiometabolic health, however the current evidence base has a number of limitations. For example, most workplace HIIT interventions to date have been conducted in university workplaces (Shepherd et al., 2015; Allison et al., 2017; Cuddy et al., 2019), which may have onsite exercise facilities (e.g., gyms and showers) that may not be available in other workplaces (Burn et al., 2020). Additionally, senior management teams in universities have a vested interest in research practices (Pinar and Unlu, 2020). As such, access to facilities and participants for research in university settings may not be generalisable to other working environments. Furthermore, the majority of trials have used cycle ergometer HIIT (Shepherd et al., 2015; Cuddy et al., 2019; Metcalfe et al., 2020), yet in employee focus groups undertaken to aid the development of the intervention described herein, a choice in a variety of exercise modes was regarded as important for enhancing engagement and enjoyment in workplace HIIT (Burn et al., 2020). Further, as choice and variety in exercise selection have been proposed as key drivers of engagement in one previous pilot workplace HIIT trial (Eather et al., 2020), using a single exercise mode across an intervention may not facilitate adherence or compliance in some individuals.

Despite the promising findings of previous pilot workplace HIIT trials, the majority of work to date has focused on intervention effectiveness in terms of changes in health or fitness outcomes. Process evaluations–a set of research activities undertaken during or after intervention implementation–can be used to explore intervention fidelity (Horner et al., 2006) and participant perspectives of the intervention (Bellg et al., 2004). Despite the wealth of information process evaluations can yield, surprisingly few workplace HIIT interventions have included process evaluations as part of the overall study design (Kinnafick et al., 2018; Metcalfe et al., 2020).

To address these limitations, there is a need for more controlled workplace HIIT trials, implemented in a variety of workplaces, which utilise a broader range of HIIT modes. Furthermore, process evaluations are required to explore participant perceptions of HIIT and intervention implementation. Accordingly, the aim of this study was to undertake a mixed-methods pilot trial of a workplace HIIT intervention named Brief Exercise at Work (BE@Work).

## Materials and Methods

### Study Design

This study used a pilot non-randomised controlled trial design. One organisation was designated as the intervention site where participants received a workplace HIIT intervention, and another separate organisation was designated as a no-intervention control group. The designation of workplaces to groups was based on the feasibility for the participating workplaces for exercise sessions to be conducted during the designated timeframe (e.g., April-July 2018).

A pilot trial is a precursor to a definitive randomised controlled trial (RCT) aiming to determine intervention effectiveness, and is undertaken to test key intervention components such as conducting outcome assessments and interventions in the settings in which they will be implemented, as well as to explore intervention fidelity and data variability (Abbott, 2014). The reporting of this study conforms with the CONSORT 2010 statement: extension for pilot and feasibility trials (Eldridge et al., 2016). Teesside University's School of Health and Social Care Research Governance and Ethics Sub-committee approved the study (study number: 036/18) and participants gave written informed consent. The protocol was prospectively registered on clinicaltrials.gov (identifier: NCT03467594).

### Participants and Recruitment

Two office-based organisations in Northeast England were invited to take part in the study. These worksites were invited based on their involvement in work undertaken specifically to design the BE@Work intervention, described elsewhere (Burn et al., 2020). In the 2 months preceding the intervention, using email distribution lists, recruitment was facilitated by the individual organisations with posters placed around the workplace and presentations during staff meetings. To allow potential participants to try the programme activities, and in-line with feedback collected during the development stage of BE@Work (Burn et al., 2020), trial HIIT sessions were conducted in the fortnight prior to baseline testing at the intervention site (Burn et al., 2020).

The BE@Work intervention was a pilot trial, of which one of the key outcomes is gaining information on baseline to follow-up random variability (Craig et al., 2008). Therefore, the target sample size which was deemed feasible for this pilot trial was 60 participants (~30 participants per study site). This target sample size reflects the fact that only one researcher was scheduled to facilitate the HIIT sessions and baseline and post-intervention testing (NB), coupled with the limited resources available for the project. Inclusion criteria were adult (aged ≥18 years) employees of the participating organisations with no health conditions that precluded them from exercise and on no medication. Exclusion criteria were symptoms of, or known presence of cardiovascular or metabolic disease, injury or co-morbidity affecting the ability to undertake exercise, early family history of sudden cardiac death, and pregnancy. All participants underwent pre-exercise screening using the Physical Activity Readiness Questionnaire for Everyone (PAR-Q+) (Bredin et al., 2013). Across the intervention and control sites, 54 participants were recruited. Thirty participants (10 males, mean [standard deviation (SD)] age: 46 ± 9 years) were recruited from the intervention site and 24 participants (10 males, mean age 46 ± 12 years) from the control site.

### Intervention Protocol

The BE@Work intervention consisted of three group-based HIIT sessions each week for 8 weeks (24 sessions in total). Exercise sessions were conducted in a meeting room or on a grassed area outside the workplace and delivered by the first author (NB, exercise science post-graduate research student with previous experience of delivering group-based exercise sessions). Based on opinions expressed by employees in focus groups during intervention development (Burn et al., 2020), multiple HIIT sessions were scheduled across the working week (19 sessions each week), with participants asked to attend any three sessions. A choice of exercise modes, which were selected by participants during intervention development, were offered both within and between sessions, based on stair climbing, stepping and boxing (Burn et al., 2020). In line with previous investigations showing a beneficial effect of HIIT (Weston K. S. et al., 2014) the target heart rate for high-intensity exercise was set at ≥85% HRmax (Weston K. S. et al., 2014) and the intensity of the prescribed exercise was quantified prior to intervention implementation. Here (Burn, 2020), participants (*n* = 15; mean ± SD age: 39 ± 11 years; body mass index 24.9 ± 3.4 kg·m^2^) conducted single sessions of HIIT based on boxing, stair climbing and stepping, with mean peak heart rates indicating high intensity work, i.e., ≥85% HR_max_ (Weston K. S. et al., 2014) (mean peak ± SD %HR_max_: box 85 ± 5 %HR_max_, step 86 ± 7 %HR_max_, stair climbing 85 ± 8%HR_max_). As there were limited differences in heart rate between modes, this indicated the modes could be used interchangeably within the intervention without compromising the exercise intensity. Example drills are detailed in Table 1.

**Table 1:** Example BE@Work HIIT drills.

**Mode**	**Example drill**
Boxing	Ten fast jabs/ upper cuts/ hooks on the focus pad and 50 m shuttle run/ power walk
	Ten fast jabs/ upper cuts/ hooks on the focus pad and 50 jumping hacks
	Ten fast jabs/ upper cuts/ hooks on the focus pad and 50 skips or jumps over a rope on the ground
Stair climbing	One stair climb (30 steps), and 100 m shuttle run/ power walk
	Repeated stair climbs (30 steps up, 30 steps down)
Stair stepping	20 step ups and downs and 20 jumping jacks or side taps
	20 steps up and down and 50 m shuttle run/ power walk

### HIIT Protocol

Based on HIIT protocols previously shown to elicit adaptations in cardiorespiratory fitness and cardiometabolic health (Hood et al., 2011; Little et al., 2011; Weston et al., 2016), the HIIT protocol consisted of four to seven 60-s high-intensity bouts, interspersed with 75-s of rest. Progression was provided by increasing the number of high-intensity bouts by one 60-s repetition every fortnight. Each session began with a 5-min warm-up and concluded with a 2-min cool down involving heart rate raising exercises relevant to the exercise session. Exercise sessions lasted ~15 min in the earlier weeks of the intervention to ≤ 22 min in the later weeks.

### Implementation Monitoring

Participants' heart rates were recorded using a validated (Rider et al., 2019) second-to-second wrist-worn monitors (Polar A360, Polar Electro, Kempele, Finland). For each participant, age predicted HR_max_ was calculated using the Tanaka equation (208-0.7^*^age in years) (Tanaka et al., 2001). If a participant exceeded this predicted value during a HIIT session, their HR_max_ was recalibrated to the higher observed value (Weston et al., 2004). Following each exercise session, individual participant heart rate files were downloaded into the Polar Flow software (Polar Electro, Kempele, Finland). The data used in the analysis was the highest 1-s value from each high-intensity bout, expressed as a percentage of the individual participant's HR_max_, across each attended HIIT session. Session ratings of perceived exertion (RPE) were recorded after the 2 min cool down, using the CR-10 scale (Borg, 1998). This scale ranges from “nothing at all” (0) to “absolute maximum” (Reed et al., 2017).

### Outcome Measures

Outcome data were collected at baseline and post-intervention from intervention and control participants (at least 72 h after and within 7 days of the final HIIT session for intervention participants). All outcomes were assessed in the participants' workplaces.

Predicted maximal oxygen uptake (VO_2max_) was estimated using the Chester step test (Sykes and Roberts, 2004) (Cartwright Fitness, Huntington, UK). Heart rate was recorded throughout the test using wrist-worn monitors (Polar A360, Polar Electro, Kempele, Finland) and was used to predict VO_2max_ using the Chester step test calculator (Cartwright Fitness, Huntington, UK). As outlined in our registered trial protocol, we had intended to conduct laboratory-based maximal aerobic exercise tests with a subsample of participants. However, management in the participating organisations highlighted that this would not be feasible, as tests needed to be conducted during work hours and participants could not be released from work to attend the university laboratory.

Leg extensor muscle power was assessed using the Nottingham leg extensor power rig (Medical Engineering Unit, University of Nottingham, Nottingham, UK). Ten maximal effort leg extensions were performed on each leg, each separated by 30 seconds of rest (Hurst et al., 2018a). The highest value was taken as the participants' peak power output for analysis. At baseline data collection, to reduce habituation effects associated with the Nottingham leg extensor power rig (Hurst et al., 2018a) it was initially intended that participants would undertake three repeated trials on the Nottingham leg rig. However, due to the significant time commitment required (~15 min per trial) the participating organisations were reluctant to allow staff extra time away from work. As a result, of the participants who consented to undertake this assessment, in the intervention group 14 and 16 participants undertook the familiarisation procedures twice and three times respectively, and in the control group 2, 15 and 5 participants undertook familiarsation procedures once, twice and three times, respectively.

Handgrip strength was measured using a hydraulic hand dynamometer (12-0240; Baseline Evaluation Instruments, Fabrication Enterprises Ltd, New York, United States). Participants performed three maximal efforts on each hand, alternating between hands each time (Perna et al., 2016). The highest value was retained for analysis.

Blood pressure was measured using an OMRON M6 AC (HEM-7322-E) monitor (Omron Healthcare UK, Milton Keynes, UK), following the European Society of Hypertension guidelines (O'Brien et al., 2010). Non-fasting blood lipids [cholesterol, triglyceride and high-density lipoprotein cholesterol (HDL-cholesterol)] and glucose were assessed using finger prick blood samples and a Cholestech LDX analyser (Cholestech Corporation, Hayward, CA, USA).

Body mass and height were measured to the nearest 0.1 kg and 0.1 cm, respectively, using the Seca 799 electronic column scale, fitted with a Seca 224 stadiometer rod (Seca, Hamburg, Germany). Body mass index (BMI) was calculated using the equation (kg/m^2^) = body mass (kg)/ height (m)^2^. Waist circumference was measured using a non-elastic Gulick tape measure with a tension device. Following normal expiration, the circumference of the abdomen was measured at the narrowest point between the lower costal border and the top of the iliac crest to the nearest 0.1 cm (Marfell-Jones et al., 2012).

Physical activity over the previous seven days was assessed using The International Physical Activity Questionnaire (IPAQ) short form (Craig et al., 2003). Scoring protocols outlined by the questionnaire developers (International Physical Activity Questionnaire, 2005) were followed to obtain a continuous physical activity score. The use of the IPAQ to assess habitual physical activity is a deviation from our registered trial protocol which stated that tri-axial accelerometers would be used. The latter were unavailable for the baseline testing period, so it was necessary to use an alternative assessment of physical activity.

Health-related quality of life (HR-QoL) was assessed using the Medical Outcomes 36-Item Short Form Health Survey 1.0 (SF-36) and scoring procedures outline by Ware et al. (2002). Possible scores range from 0 to 100 Arbitrary Units (AU) with higher scores indicating higher HR-QoL. Mental well-being was assessed using the Warwick Edinburgh Mental Well-being Scale (WEMWS) and scoring procedures outlined by Tennant et al. (2007). Scores range from 14 AU to 70 AU and higher scores indicate positive mental well-being. Perceived stress was measured using the Perceived Stress Scale (PSS) (Cohen et al., 1995). Scoring followed procedures outlined by Cohen et al. (1995) and possible scores range from 0 AU to 40 AU with lower scores indicating lower perceived stress.

### Post-intervention Focus Groups

During the post-intervention testing period, intervention participants were invited, via email, to attend a focus group. Focus groups provided an opportunity to elicit opinions via group discussion rather than individual reflection (Kitzinger, 1995) and also mirror the group-based nature of the BE@Work sessions. The focus groups were designed to explore participants' perceptions of the BE@Work intervention. Nine intervention participants (3 male, mean [± SD] age: 48 [± 7] years, session attendance 21 ± 2 sessions) volunteered to attend one of three focus groups. Focus groups consisted of two to four participants and were facilitated by one study author (KLW). Each focus group lasted between 48 and 59 min and were audio recorded and transcribed verbatim resulting in 93 pages of data (Arial, font size 12, 1.5 line spacing).

### Statistical Analysis

Heart rate data were used to explore intervention fidelity [i.e., whether the intervention was delivered as intended in a comparable manner to all participants (Dumas et al., 2001)]. The proportion of completed repetitions over the intervention in which the high-intensity exercise criterion was attained [≥85%HR_max_ (Weston K. S. et al., 2014)], was determined for each participant (Taylor et al., 2015). The median and interquartile range of these proportions was calculated. Then, to provide the correct overall between and within-subject variability (expressed as a standard deviation [SD]) in peak heart rate across the high-intensity bouts, a linear mixed model was applied, with exercise session specified as a fixed effect (SPSS v.25, Armonk, NY: IBM Corp). A similar process was used to analyse RPE data, which was included to further illustrate exercise intensity using a potentially more cost effective and scalable tool than heart rate monitors.

To inform the sample size for a future RCT, change variance was estimated for predicted VO_2max_ (Abbott, 2014). With the primary aim of piloting the data analysis that would be appropriate for a full RCT, we analysed our outcome data using an analysis of covariance (ANCOVA) model (SPSS v.25, Armonk, NY: IBM Corp) (Vickers, 2005). Plots of the model residuals vs. the predicted values were visually inspected to check they were correctly specified (uniform variance and normal distribution of residuals). The fixed effect was the group (intervention or control), and the dependent variable was the change from baseline to post-intervention. Model covariates were sex, age and baseline value of the outcome, to adjust for any imbalances between the intervention and control groups at baseline (Vickers and Altman, 2001). For the blood lipid and glucose measures, fasting status was included as an additional covariate. This variable was defined as the number of hours fasted post-intervention minus number of hours fasted at baseline. Data are expressed as mean ± SD, with uncertainty in all estimates expressed as 95% confidence intervals (CI). In keeping with guidance for the analysis of pilot trials (Lancaster et al., 2004), Cohens d effect sizes (Cohen, 1988) were derived and findings were interpreted as follows; trivial <0.2, small 0.2–0.3, moderate 0.4–0.8, and large >0.8. We did not undertake null hypothesis testing or report *P*-values (neither absolute or Bonferroni-corrected), in keeping with reporting guidance for exploratory pilot studies (Lancaster et al., 2004; Lee et al., 2014).

### Focus Group Data Analysis

Focus group data were analysed using directed content analysis (Hsieh and Shannon, 2005) where the structure of the analysis is informed by previous research or theory (Elo and Kyngäs, 2008) using NVivo 12. As the purpose of the focus groups was to explore participants' perspectives of the BE@Work intervention, a pre-defined categorisation matrix was developed focusing on important intervention elements (Table 2). Following transcription and re-reading of the transcripts, two authors (NB and MG) coded the transcripts based on the pre-determined categorisation matrix. These authors examined the data independently within the pre-defined categories and inductively coded to further explore participant perceptions of each intervention element. During this inductive coding, NB and MG discussed coding decisions, and iteratively developed sub-categories within the pre-defined categories. MG was blinded to BE@Work programme aims during qualitative data analysis.

**Table 2:** Focus group categorisation matrix.

**Category**	**Description**
Barriers	Opinions of barriers to BE@Work session attendance (not related to intervention structure components)
Facilitators	Opinions of facilitators for BE@Work session attendance (not related to intervention structure components)
Intervention structure: Frequency	Opinions of the acceptability and feasibility of the frequency of BE@Work exercise sessions
Intervention structure: Timing of exercise sessions	Opinions of the acceptability and feasibility of the time of day BE@Work exercise sessions were offered
Intervention structure: Length of exercise sessions	Opinions of the acceptability and feasibility of the length of BE@Work exercise sessions
Intervention structure: Length of the intervention	Opinions of the acceptability and feasibility of the length of the BE@Work intervention
HIIT	Opinions of the acceptability and feasibility of high-intensity interval training in the workplace!!break Experiences of HIIT
Exercise modes	Opinions of the acceptability and feasibility of BE@Work exercise modes (boxing, stepping, stair climbing, shuttle runs, skipping)
Overall programme considerations: Group based HIT sessions	Opinions of the acceptability and feasibility of the group based nature of BE@Work exercise sessions
Overall programme considerations: Location of exercise	Opinions of the acceptability and feasibility of the location of BE@Work exercise sessions

## Results

**Figure 1:**
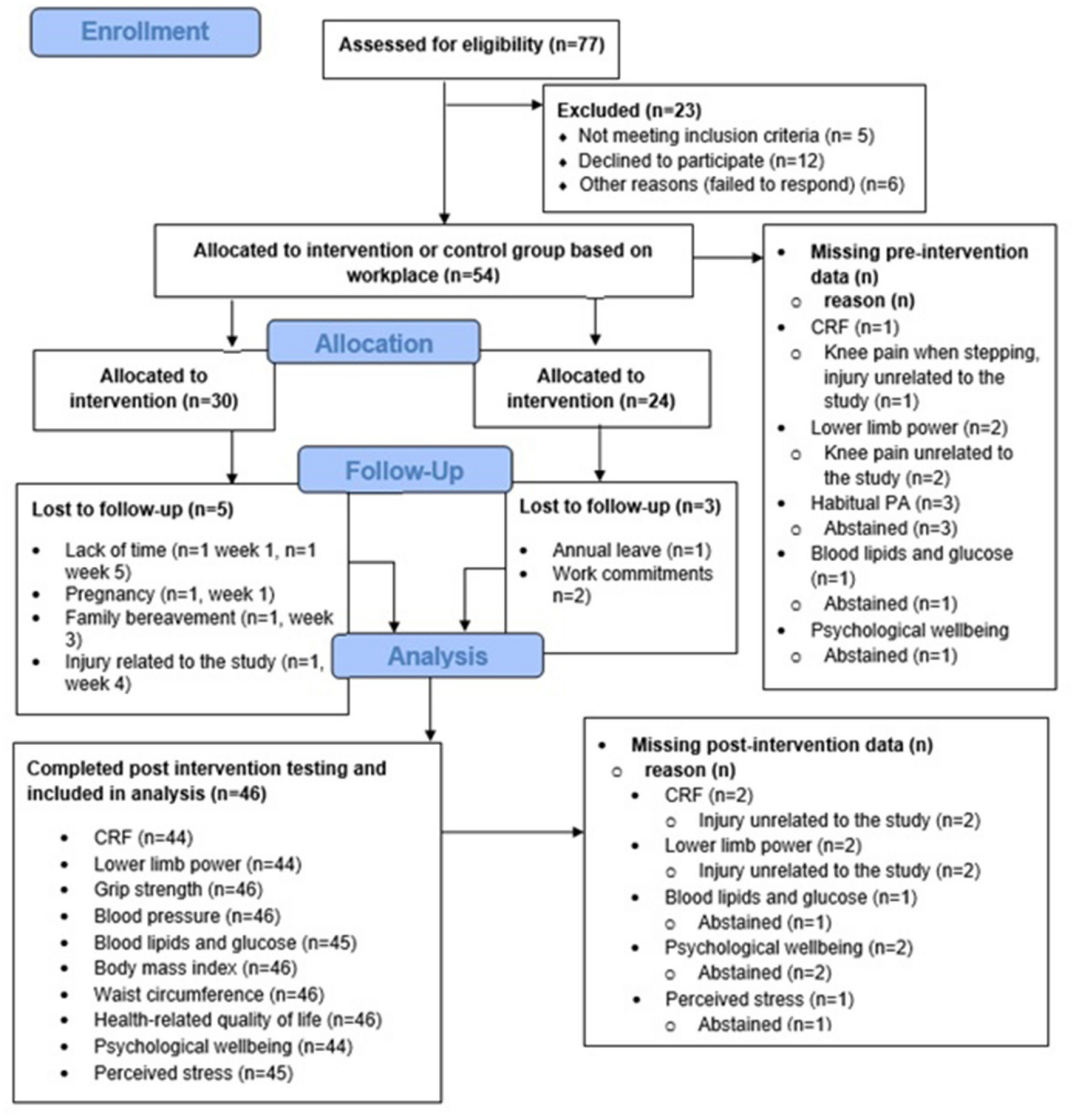
CONSORT 2010 flow diagram (Eldridge et al., 2016).

Participant flow through the study is shown in Figure 1. Baseline participant characteristics are shown in Table 3. Of the 54 participants that were allocated to the intervention or control group, 25 of 30 intervention and 21 of 24 control participants completed post-intervention testing. Reasons for drop-out in the intervention group included pregnancy (*n* = 1), lack of time (*n* = 2), family bereavement (*n* = 1) and injury related to the study (*n* = 1).

**Table 3:** Baseline participant characteristics (mean ±SD).

	**Intervention** ** (*n* = 30)** ** mean (±SD)**	**Control (*n* = 24)** ** mean (±SD)**
Age (years)	46 (±9)	46 (±12)
Sex (male/female)	10/20	10/14
Ethnicity	White British (*n* = 29) Asian or Asian British (*n* = 1)	White British (*n* = 24)
Height (m)	1.7 (±0.1)	1.7 (±0.1)
Body mass (kg)	77.0 (±15.0)	74.1 (±10.1)
Body mass index (kg/m^2^)	27.0 (±4.7)	26.2 (±3.2)
Habitual physical activity (METmin^−1^)	1,354 (±819)	2,006 (±1,695)

### Feasibility

#### Attendance

Of the 25 participants who completed the intervention, mean ± SD session attendance was 20 ± 3 out of 24 possible sessions (82 ± 15%). Reasons for non-attendance included work and family commitments, annual leave from work, injuries unrelated to the study, and in two participants, injuries related to the intervention.

#### Injuries Related to the Intervention

Two injuries attributable to the intervention occurred during BE@Work. One participant tripped while completing a shuttle run, resulting in a fractured elbow. The participant withdrew from the study, but subsequently made a full recovery. One participant suffered a minor calf strain during a shuttle run which resulted in one missed session.

#### Implementation and Intensity Quantification

Individual participant heart rate traces were available from 94% of attended HIIT sessions. This resulted in 506 heart rate data files, compromising 2,735 high-intensity bouts. Of the high-intensity bouts recorded over the intervention, the median (interquartile range) proportion of completed repetitions where heart rate corresponded to the criterion for high-intensity work (≥85%HR_max_) was 70% (53–84%). The mean ± SD peak heart rate across the intervention was 87% HR_max_ with a within-subjects SD of 5.5% (95% CI: 5.3–5.6%) and between-subjects SD of 3.7% (95% CI: 2.8–4.8%).

From the attended HIIT sessions, 526 individual session RPEs were recorded. Mean session RPE over the intervention was 6 AU (between the qualitative descriptors of “hard” and “very hard” on the CR-10 scale), with a within-subjects SD of 1.3 AU (95% CI: 1.3–1.4 AU) and between subjects SD of 1.4 AU (95% CI: 1.1 to 1.9 AU).

### Preliminary Effectiveness

The SD of baseline to follow-up change was 6.6 mL·kg^−1^·min^−1^ (95% CI: 5.5 to 8.3 mL·kg^−1^·min^−1^) across both study groups. This change variance can be incorporated into a sample size estimation for a RCT. Assuming a clinically important mean change of 3.5 mL·kg^−1^·min^−1^ (Lee et al., 2011) and a statistical power of 90%, it was estimated using GPower that 76 participants would be required in each study group for a non-clustered RCT. If, in a future trial, researchers recruit participants in a specific clustered fashion, then this should be considered in any future sample size estimation, depending on the specific cluster sizes and the intraclass correlation between clusters.

Baseline values and effect statistics for the pilot between-group comparisons for physical fitness, cardiometabolic health and mental well-being outcomes are presented in Tables 4–6. After adjusting for age, sex and baseline values, compared with control the intervention effect on VO_2max_ was a moderate effect of 3.9 mL·kg^−1^·min^−1^ (95%CI: −0.2 to 8.1 mL·kg^−1^·min^−1^, *d* = 0.47). When considering all other outcome measures, we observed moderate effects for two domains of HR-QoL (pain; −6.7 AU; −18.4–5.0 AU, d = −0.67 and vitality 8.7 AU; 1.7–15.8 AU, d = 0.51), and perceived stress (-2.6 AU; −5.7 to 0.4 AU, d = −0.4). We observed small effects for one domain of HR-QoL (general health perceptions 4.8 AU; −0.3 to 9.7 AU, d = 0.35), mental well-being (2.5 AU; −0.8 to 5.7, d = 0.34), physical activity (−310 MET.min.week^−1^: −1,640 to 1,019 MET.min.week^−1^, d = −0.25), non-dominant leg extensor muscle power (15.9 Watts; 1.6–30.1 Watts, d = 0.2) and HDL-cholesterol (−0.1 mmol.L^−1^; −0.2 to 0.04 mmol.L^−1^, d = −0.2).

**Table 4:** Intervention effect on physical fitness variables.

	**Intervention** ** (BE@Work;** ***n*** **=** **25)**		**Control** ** (CON;** ***n*** **=** **21)**		**Between group comparison** ** (BE@Work–CON)**	
	**Baseline value** ** (mean ± SD)**	**Adjusted mean change** ** (mean [95%CI])**	**Baseline value** ** (mean ± SD)**	**Adjusted mean** ** change** ** (mean [95%CI])**	**Adjusted mean** ** change** ** (mean [95%CI])**	**Effect size**
VO_2max_ (mL·kg^−1^·min^−1^)	37.7 ± 7.5	4.7 [2.1 to 7.4]	36.6.0 ± 9.0	0.8 [−2.3 to 3.9]	3.9 [−0.2 to 8.1]	0.47
Leg extensor muscle power (non-dominant leg, watts)	212.0 ± 69.9	−13.0 [−22.6 to −3.4]	236.6 ± 89.6	−31.5 [−39.3 to −18.5]	15.9 [1.6 to 30.1]	0.20
Leg extensor muscle power (dominant leg, watts)	199.3 ± 66.6	−2.6 [13.5 to 8.4]	230.0 ± 95.5	−19.6 [−31.7 to −7.5]	12.1 [−3.5 to 27.7]	0.15
Hand grip strength (dominant hand, kg)	35.6 ± 10.4	0.2 [−1.3 to 1.7]	38.1 ± 9.2	1.4 [−0.2 to 3.0]	−1.2 [−3.4 to 0.9]	−0.12
Hand grip strength (non-dominant hand, kg)	33.6 ± 10.7	0.5 [−0.6 to 1.5]	36.6 ± 9.0	−0.3 [−1.5 to 0.9]	0.8 [−0.8 to 2.4]	0.08

**Table 5:** Intervention effect on cardiometabolic health variables.

	**Intervention** ** (BE@Work;** ***n*** **=** **25)**		**Control** ** (CON;** ***n*** **=** **21)**		**Between group comparison** ** (BE@Work–CON)**	
	**Baseline value** ** (mean ± SD)**	**Adjusted mean change** ** (mean [95%CI])**	**Baseline value** ** (mean ± SD)**	**Adjusted mean change** ** (mean [95% CI])**	**Adjusted mean change** ** (mean [95% CI])**	**Effect size**
Habitual physical activity (MET.min.week${}_{-}^{1}$)	1,371 ± 831	635 [−246 to 1,517]	2,034 ± 1,695	946 [−19 to 1,911]	−310 [−1,640 to 1,019]	−0.25
HDL cholesterol (mmol.L^−1^)	1.6 ± 0.5	−0.1 [−0.2 to −0.03]	1.6 ± 0.5	−0.03 [−0.1 to 0.1]	−0.1 [−0.2 to 0.04]	−0.20
Glucose (mmol.L^−1^)	5.2 ± 0.6	0.2 [−0.2 to 0.7]	5.5 ± 0.9	0.2 [−0.3 to 0.6]	0.09 [−0.5 to 0.7]	0.12
Systolic blood pressure (mmHg)	128 ± 12	−6.8 [−10.4 to −3.2]	135 ± 14	−8.0 [−11.9 to −4.1]	1.2 [−4.3 to 6.7]	0.09
Triglycerides (mmol.L^−1^)	1.2 ± 0.7	−0.03 [−0.2 to 0.2]	1.4 ± 0.6	−0.1 [−0.3 to 0.1]	0.06 [−0.2 to 0.3]	0.09
Waist circumference (cm)	86.2 ± 11.9	−0.3 [−1.1 to 0.5]	85.4 ± 8.7	−0.1 [−1.0 to 0.7]	−0.2 [−1.4 to 1.0]	−0.02
Diastolic Blood Pressure (mmHg)	80 ± 7	−2.5 [−5.2 to 0.1]	86 ± 8	−2.6 [−5.5 to 0.2]	0.1 [−3.9 to 4.1]	0.01
Total cholesterol (mmol.L^−1^)	5.5 ± 0.9	−0.2 [−0.4 to 0.04]	5.4 ± 1.0	−0.2 [−0.5 to 0.1]	0.007 [−0.3 to 0.4]	0.01
Body mass index (kg.m^2^)	26.8 ± 4.6	−0.07 [−0.4 to 0.2]	26.2 ± 3.2	−0.05 [−0.4 to 0.3]	−0.02 [−0.5 to 0.4]	−0.01

**Table 6:** Intervention effect on mental well-being variables.

	**Intervention** ** (BE@Work;** ***n*** **=** **25)**		**Control** ** (CON;** ***n*** **=** **21)**		**Between group comparison** ** (BE@Work–CON)**	
	**Baseline value** ** (mean ± SD)**	**Adjusted mean change** ** (mean ± 95%CI)**	**Baseline value** ** (mean ± SD)**	**Adjusted mean change** ** (mean ± 95% CI)**	**Adjusted mean change** ** (mean; ±95% CI)**	**Effect size**
HR-QoL: Pain (AU)	90.8 ± 10.0	−11.7 [19.6 to −3.9]	91.1 ± 10.1	−5.0 [−13.7 to 3.5]	−6.7 [−18.4 to 5.0]	−0.67
HR-QoL: vitality (AU)	61.6 ± 18.1	11.5 [6.7 to 16.1]	62.8 ± 16.3	2.6 [−2.5 to 7.8]	8.7 [1.7 to 15.8]	0.51
Perceived stress scale (PSS) (AU)	13.4 ± 6.7	−2.9 [−4.9 to −0.8]	12.9 ± 6.2	−0.2 [−2.4 to 2.0]	−2.6 [−5.7 to 0.4]	−0.40
HR-QoL: General health perceptions (AU)	66.3 ± 14.9	3.9 [0.6 to 7.3]	65.5 ± 12.2	−0.8 [−4.5 to 2.8]	4.8 [−0.3 to 9.7]	0.35
Warwick Edinburgh mental well-being scale (WEMWS) (AU)	50.4 ± 8.1	5.8 [3.6 0 to 8.0]	52.0 ± 6.4	3.3 [0.9 to 5.7]	2.5 [−0.8 to 5.7]	0.34
HR-QoL: Emotional well-being (AU)	75.8 ± 13.7	4.7 [0.05 to 9.4]	77.3 ± 13.3	2.9 [−2.2 to 8.1]	1.8 [−5.2 to 8.8]	0.13
HR QoL: Role limitations due to physical health problems (AU)	97.0 ± 11.0	−8.6 [−20.9 to 3.7]	95.2 ± 15.0	−10.0 [−23.5 to 3.5]	1.4 [−17.0 to 19.8]	0.11
HR QoL: Role limitations due to emotional health problems (AU)	88.0 ± 28.7	0.2 [−10.7 to 11.2]	90.5 ± 26.1	−0.3 [−12.3 to 11.7]	0.5 [−15.9 to 16.8]	0.02
HR QoL: Physical functioning (AU)	93.6 ± 14.4	1.0 [−1.4 to 3.4]	96.7 ± 4.3	1.0 [−1.2 to 4.1]	0.03 [−3.6 to 3.7]	0.00
HR-QoL: Social functioning (AU)	88.0 ± 17.5	−0.6 [−8.3 to 7.1]	89.4 ± 13.8	−0.6 [−9.0 to 7.8]	0.05 [−11.4 to 11.5]	0.00

*Key: HR-QoL possible scores: 0–100 AU, WEMWS possible scores: 14–70 AU, PSS possible scores: 0–40 AU*.

### Acceptability

The following section describes the findings of the post-intervention focus groups, with illustrative participant quotes.

#### Intervention Logistics

##### Frequency of HIIT Sessions

Most participants reported that thrice weekly HIIT sessions were satisfactory. Some participants reported daily HIIT sessions would be too time consuming, whereas others reported that once weekly HIIT sessions could negatively impact engagement “*I think one [session per week] is not often enough really.you could also lose interest*” (Focus Group 2).

##### Timing of HIIT Sessions

In the BE@Work trial, a flexible schedule of HIIT sessions were offered throughout the week. While there was no consensus amongst participants as to the most appropriate time of day for HIIT sessions (e.g., before work, midday or after work), the flexible nature of the HIIT sessions were particularly important for enhancing attendance. The participants could not identify any single session that was universally poorly attended, highlighting the perceived importance of flexible session attendance “*I felt there was enough times [HIIT sessions] to suit everyone. And I think everyone used all the different times” (*Focus Group 2). In particular, participants noted that being able to change session times allowed them to fit HIIT around work requirements “*Occasionally, I would say “I'm really sorry, I can't come to this one [scheduled HIIT session] but I will come to another one” and she [exercise facilitator] was absolutely fine. A lot of flexibility*” (Focus Group 1).

##### Length of HIIT Sessions

Participants regarded the short session length ( ≤ 22 min) as particularly important. Short sessions accommodated their work commitments, and it was also important that the sessions did not overrun or begin late “*I mean we always got back within the half hour. So you never overran. It doesn't matter how many people turned up for the group, you just knew you'd be back at your desk within half an hour.”* (Focus Group 2).

##### Exercise Modes

While there was no consensus amongst participants as to favoured exercise modes (stair climbing, stepping or boxing), a choice in a variety of exercise modes maintained interest and engagement:

“*And the fact that she chunked it up so when we had seven minutes, we'd have like two of boxing and then when you've done that once you think right one more and then you move on to something else. So that made it a lot easier. Whereas, if it had have been 7 min of boxing I'd have lost the will to live.”* (Focus Group 1).

##### Group-Based

The group-based nature of the BE@Work intervention was perceived positively. While some participants reported initially feeling anxious or self-conscious about exercising with colleagues, over the intervention these feelings diminished:

“*It was because I knew it was gonna (sic) be with a person that I had to work with. So like, if it's working in partners it does make me a bit anxious. Whereas, it wasn't [as bad as expected] and that's what I was saying.everyone was in the same boat.”* (Focus Group 2).

Group-based HIIT sessions facilitated relationships with colleagues and camaraderie, which was deemed an important benefit of the intervention:

“*There's been the banter that's been about that [the BE@Work intervention], the talks that you've had about it and I think that's really helped me through, to give me a bit of a better head really. To go “Oh are you going today?” “Yeah, I'm going, I'll walk over with you”* (Focus Group 2).

##### Location

The participating workplace had limited room availability for indoor exercise, therefore 70% of BE@Work sessions were conducted in the outdoor space close to the workplace. Some participants reported apprehension around outdoor exercise:

“*I wasn't sure where she was going to do it [HIIT sessions], and I had visions of us [exercising] in the car park and I was thinking this is going to be a nightmare in front of everybody. And then when we went to the gardens and that was great and then after that I wasn't bothered who saw me.”* (Focus Group 1).

Participants reported that the indoor meeting room used for HIIT sessions was too warm and not suitable for exercise. Outdoor sessions were preferred because participants enjoyed the fresh air and a break from the office environment “*But we also had a choice between inside and outside. And to begin with, I thought I wanted to be inside, but actually I much preferred outside. You kept a lot cooler…at least you got fresh air”* (Focus Group 3).

##### Exercise Facilitator

Participants reported that they enjoyed being “coached” through the HIIT sessions by an exercise facilitator. This was deemed particularly important for boxing, where participants reported that using the correct technique was important:

“*I think it's things like that, little tips [from the facilitator], little bits and it does drive you on. So the next time I did it, I made sure I did it right [boxing technique]…I was thinking she's going to be watching out for me.”* (Focus Group 2).

It was important for participants that the exercise facilitator was personable, gave clear instructions, was encouraging and was aware of the participants' limitations or exercise preferences:

“*I thought she handled a group of older people very very well really. She was always clear in her instructions. It was never condescending, she was supportive and egged you on, really sort of motivated you… and I thought her communication was good. You always knew in advance what was happening*.” (Focus Group 2).

#### Experiences of HIIT

The following section describes participant experiences of HIIT. The subcategories which were developed within this category were “physiological responses,” “psychological responses,” “rest breaks” and “relative intensity.”

##### Physiological Responses

Participants described their physiological responses to HIIT in terms of central exertion such as breathlessness and increased heart rate “*when you're at the end [of an interval] going like* ^*^mimics breathing heavily^*^” (Focus Group 3) and local muscular fatigue “*It was intense and I felt my legs hurt and I got a couple of stitches as I was doing it”* (Focus Group 2). HIIT also resulted in increased temperature and sweating “*I definitely felt hot, that my heart rate was increasing yeah and tired sometimes”* (Focus Group 2). These responses were generally viewed as acutely unpleasant.

##### Psychological Responses

Despite reporting high effort and tiredness during intervals, participants described that HIIT made them feel alert “*[HIIT makes you feel] alert I think, because you are fully concentrating”* (Focus Group 2). One participant said that HIIT made them feel energetic “*At that time of night [after work session]…you're tired, and you go [to the HIIT session] you would come alive, I guess from doing some exercise” (*Focus Group 3). Many participants reported that overall they enjoyed HIIT and found it interesting:

“*I liked it, it was the first time I had really done it [HIIT]. And yeah, I really enjoyed it. I guess it really encouraged me to do more of that sort of thing. I think when you've only got a short period of time… I think it was good”* (Focus Group 3).

##### Rest Breaks

The rest breaks incorporated into the BE@Work HIIT protocol were perceived as particularly important to make the intensity of the exercise achievable “*It was only that minute and a bit rest that made it…achievable”* (Focus Group 1) and because the breaks allowed participants to sustain high-intensity exercise throughout the HIIT session “*I think the time was good. You know, I mean you did push yourself for the minute. Because in your head you can manage it”* (Focus Group 2).

##### Relative Intensity

Participants reported that the relative intensity or “*individual nature*” of HIIT was particularly important, such that HIIT can be enacted differently in different individuals depending on their fitness level. The excerpt below details a discussion between participants from Focus Group 1, where they discuss that the relative intensity of HIIT can facilitate engagement by making it an inclusive activity:

Participant: “*You can be really unfit or you could be really fit. And it doesn't matter”*
Researcher: “*And why doesn't it matter?”*
Participant: “*Because you're not in a competition”*
Participant: “*You're only in competition with yourself not with anybody else”*
Participant: “*And you get out of it what you put in and you work to the best of your ability”*
Participant: “*I think that's the best bit really”*

#### Perceptions of Outcomes

The following section describes participant perceptions of the effects of the intervention. The subcategories which were developed within this category were “physical fitness,” “mental well-being” and “behaviour change.”

##### Physical Fitness

Participants reported that they “*felt fitter,”* because they could complete more HIIT intervals at the end of the intervention than at the beginning “*Initially we did four [intervals] and I think if we had to do seven [intervals] to begin with, I don't think I would have managed seven. Whereas, I managed seven at the end of 8 weeks, so it must have improved my fitness” (*Focus Group 1), or because they noted improvements in other activities “*So I've been doing the [community 5 k run] since January, and in the 8-week period that this has been happening [BE@Work intervention] they have been much easier to do than beforehand”* (Focus Group 2).

##### Mental Well-being

Participants reported that HIIT sessions provided a distraction from daily life “*I do think it gives you time to clear your head. That's one of the good things. It's only a short amount of time but it really does take your head out of the game a little bit” (*Focus Group 2). Participants also reported feeling more positive after a HIIT session “*I just felt happier and more energised afterward. So yeah, I felt like it was doing me some good”* (Focus Group 3). Some participants also noted reductions in their perceived stress levels “*I was really stressed before I started, but within a couple of weeks of doing this I felt much more like my usual self. I used to really look forward to doing them [the HIIT sessions]. So I'm sure this helped”* (Focus Group 3).

##### Behaviour Change

Some participants reported intentions to modify their physical activity behaviours following BE@Work. These intentions were associated with enjoying specific exercise modes “*It was the boxing. I'm actually going to try and find somewhere that does it because I really enjoyed it”* (Focus Group 1), or the use of facilities nearby to the workplace:

“*I could say “why don't we all get together and go and run around that bridge?” You didn't need a researcher there to do that. She used a lot of the stuff [exercise facilities] that's just out there [near the workplace]. It was just using the steps that are actually out there and that bit of grass has never been used as much in its life”* (Focus Group 2).

## Discussion

While a preliminary body of evidence suggests that workplace HIIT may elicit improvements in markers of physical fitness and cardiometabolic health (Shepherd et al., 2015; Allison et al., 2017; Cuddy et al., 2019; Eather et al., 2020; Metcalfe et al., 2020), most trials to date have been implemented in university workplaces, using a single exercise mode and have not explored participant perceptions of the intervention. To help explore the effectiveness of workplace HIIT further, we undertook a mixed-methods exploratory pilot trial of a workplace HIIT intervention.

### Feasibility

Notwithstanding any potential adaptations induced by exercise training, to promote long term adherence and compliance, it is essential that interventions are practical and feasible for participants. In the BE@Work trial, mean HIIT session attendance was 83%, which is similar to reported attendance in previous workplace HIIT interventions [range 71% (Eather et al., 2020) to 99% (Allison et al., 2017)]. While high session attendance could indicate sampling bias as discussed subsequently, to promote session attendance in BE@Work, a flexible approach was taken to exercise session attendance; such that 19 group HIIT sessions were conducted each week, with participants asked to attend any three sessions per week. Although this may have promoted attendance, the scalability of providing this volume of exercise sessions across a week is questionable. Indeed, the cost of employing exercise facilitators may inhibit organisations from being able to provide such flexible services to employees.

While useful, session attendance does not provide information regarding the extent to which the intervention was delivered as intended in a comparable manner to all participants; otherwise known as the intervention fidelity (Taylor et al., 2015). We used exercise intensity as an indicator of intervention fidelity. Across the intervention, the median proportion (interquartile range) of repetitions where the high-intensity exercise criterion (≥85% of HR_max_) was attained was 70% (53–84%). Previous evaluations of intervention fidelity in HIIT trials with older adults (Hurst et al., 2018b) and adolescents (Taylor et al., 2015) have categorised high-intensity exercise criterion attainment proportions of between 58 and 62% of repetitions as “moderate” intervention fidelity. With this in mind and using proposed intervention fidelity thresholds of <50% “low”; 50–70% “moderate” and >70% “high” (Hurst et al., 2018b), the intervention implementation in BE@Work could be described as moderate to high. Across the high-intensity bouts performed across BE@Work, the mean peak heart rate was 87% HR_max._ By adding and subtracting the within-subject variability to the mean peak heart rate (5.5 percentage points), this shows the intensity of the intervention ranged between 81.5% HR_max_ through to 92.5% HR_max_ in individual participants across the intervention. Furthermore, the relatively small between-subjects SD (3.7 percentage points of the mean peak heart rate) indicates that the intensity was similar between the participants (83.3% HR_max_ to 90.7% HR_max_). These findings indicate the quality of the delivery of the BE@Work intervention, and its receipt and enactment by the participants (Taylor et al., 2015). The mean peak heart rate across BE@Work was however, lower than the mean peak heart rate reported in previous community [95 ±3% HR_max_ (Reljic et al., 2018)] and workplace [91 ±3% HR_max_ (Shepherd et al., 2015)] HIIT trials. Although between-study differences are likely multifaceted, one possible explanation could be differences in exercise modalities (Buchheit and Laursen, 2013). Previous trials (Shepherd et al., 2015; Reljic et al., 2018) exclusively used cycle ergometers as the HIIT mode which can be manipulated to control exercise intensity. The BE@Work intervention used a range of exercise modes, which may explain the different exercise intensity. In regard to rating of perceived exertion, the mean session RPE was 6 AU across the intervention, which is between the qualitative descriptors of “hard” and “very hard.” This is similar to that reported in a previous workplace HIIT trial (mean RPE 16 AU, “very hard” using the 6 to 20 RPE scale) (Allison et al., 2017). Similar to the heart rate data, the relatively small within and between-subjects SDs (1.3 AU and 1.4 AU, respectively) indicate that perceived exertion of the HIIT sessions was relatively uniform both across the intervention and between participants. The within and between subject variability demonstrate that RPE ranged from 4.7 AU (i.e., ~hard) to 7.3 AU (i.e., very hard) across the intervention, and between 4.6 AU and 7.4 AU between participants.

### Preliminary Effectiveness

Post-intervention, we observed preliminary evidence of a moderate effect on VO_2max_ (3.9 mL·kg^−1^·min^−1^; −0.2 to 8.1 mL·kg^−1^·min^−1^, d = 0.47) in intervention participants compared to controls. This improvement is similar to the 2.8 to 3.5 mL·kg^−1^·min^−1^ increases in VO_2max_ reported following previous workplace HIIT trials (Shepherd et al., 2015; Allison et al., 2017; Cuddy et al., 2019). Although the effect we observed is higher than that reported in a previous meta-analysis of workplace exercise interventions (2.7 mL·kg^−1^·min^−1^) (Burn et al., 2019), it is lower than the increase in VO_2max_ reported following largely laboratory-based HIIT trials (5.5 mL·kg^−1^·min^−1^) (Milanovic et al., 2015). This could be because the magnitude of intervention effects are attenuated and more variable under less tightly controlled conditions outside of the laboratory (Courneya, 2010). Our somewhat larger mean treatment effect in this small pilot trial could also be due to sampling error, a phenomenon that is sometimes referred to as the “winners curse” (Tugwell and Knottnerus, 2018). Therefore, we also sought to use the SD of baseline to follow-up change in VO_2max_ (6.6 mL·kg^−1^·min^−1^) to estimate a sample size for a full RCT. Assuming a clinically important mean change of 3.5 mL·kg^−1^·min^−1^ (Lee et al., 2011), 76 participants would be required in each study group to detect this mean change. Of the previous workplace HIIT trials, sample sizes have ranged from 13 (Metcalfe et al., 2020) to 42 (Shepherd et al., 2015) participants in the HIIT group. This may highlight the difficulty in acquiring funding, conducting, or recruiting participants in the workplace to conduct a fully powered trial. It is important to note however, that our findings may have been influenced by sampling bias [e.g., when a sample does not accurately reflect the true target population (Lavrakas, 2008)]. In the case of the BE@Work intervention, it could be that individuals who volunteer for workplace HIIT studies differ from the working age population. Indeed, when comparing our participants' baseline fitness levels to normative reference values for apparently healthy middle-aged British adults (Ingle et al., 2020), the baseline VO_2max_ for males in our sample (42.7 mL·kg^−1^·min^−1^) was higher than the British median (36.9 mL·kg^−1^·min^−1^). Nonetheless, baseline VO_2max_ for women in our sample (33.5 mL·kg^−1^·min^−1^) was lower than the British median (36.5mL·kg^−1^·min^−1^).

It is concerning that we observed a moderate increase (indicated by a decrease in HR-QoL score) in pain (−6.7 AU; −18.4 to 5.0 AU, *d* = −0.67. An increase in perceived pain has been reported following one previous community-based HIIT trial in inactive and overweight adults (Lunt et al., 2014). Given that critiques of HIIT have highlighted that perceived negative experiences, such as perceived pain during exercise may impact participation in the activity (Biddle and Batterham, 2015), this area is worthy of further investigation. Interestingly, while increases in pain were not explicitly reported by our focus group participants, they did report local muscular fatigue during HIIT. As the SF-36 does not differentiate between acute or chronic pain or type of pain, it could be that sensations of exertion influenced their perception of pain when completing the SF-36, however this requires further investigation.

We observed moderate improvements in vitality which is one domain of the SF-36 HR-QoL questionnaire (8.7 AU; 1.7 to 15.8 AU, *d* = 0.51). Vitality is the balance an individual feels between energy and fatigue (Ware et al., 2002). Both workplace HIIT specifically (Shepherd et al., 2015) and exercise training more generally, have been shown to improve markers of vitality (Puetz et al., 2006). However, because social interaction during exercise may improve vitality more than exercising alone (McNeil et al., 1991), while the increase in vitality observed in our study could be attributed to HIIT, it could also be attributed to the group-based nature of the intervention. We also observed a moderate decrease in perceived stress (−2.6 AU; −5.7 to 0.4 AU, *d* = −0.4) post-intervention. While perceived stress has not been assessed in previous workplace HIIT trials, improvements in this outcome could be particularly meaningful for employers because work-stress is associated with both absenteeism (Lauzier et al., 2017) and productivity (Hoboubi et al., 2017). Of the other well-being outcomes assessed, we observed a small effect on one domain of HR-QoL assessed using the SF-36 (general health perceptions: 4.8 AU; −0.3 to 9.7 AU, *d* = 0.35). This finding is supported by previous community-based HIIT trials, where improvements in quality of life have also been reported (Lunt et al., 2014; Shepherd et al., 2015). Furthermore, we observed small improvements in mental well-being assessed via the WEMWS (2.5 AU;−0.8 to 5.7, d= 0.34). This finding is supported by a previous systematic review indicating that workplace exercise interventions can improve well-being (Abdin et al., 2018).

Compared with controls, we observed a small reduction in self-reported physical activity (-310 MET.min.week^−1^: −1,640 to 1,019 MET.min.week^−1^, *d* = −0.25) in the intervention group. While within-group comparisons showed increases in physical activity in both groups, the increase was more pronounced in the control group and therefore the intervention effect was a reduction in physical activity. Given that previous HIIT interventions have observed increases in physical activity in inactive adults (Stavrinou et al., 2019) and the validity and reliability of self-report measures of physical activity have been questioned (Prince et al., 2008), future studies could seek to use device-based measures of physical activity to further explore the effect of workplace HIIT on habitual physical activity.

We observed evidence of a small increase in leg extensor muscle power (non-dominant leg extensor muscle power; 15.9 Watts: 1.6–30.1 Watts, *d* = 0.2), compared with controls. However, within-group comparisons showed decreases in muscle power in both groups, with the reduction more pronounced in the control group. The mechanism for a reduction in muscle power in both groups is unclear but could be related to habituation effects associated with the Nottingham leg extensor power rig (Hurst et al., 2018a). While contemporary guidance does not require familiarisation protocols to be conducted post-intervention (Hurst et al., 2018b), it is possible that these findings are a result of insufficient familiarisation at baseline as previously described or participants may have benefitted from further familiarisation, post-intervention. In comparison to previous work, while improvements in muscular power measured via standing long jump have been observed in one previous workplace HIIT study (Eather et al., 2020), a meta-analysis (Weston, M. et al., 2014) and HIIT trials in adults (Greenlee et al., 2017; Eather et al., 2019) have not observed changes in muscular power. Despite these conflicting findings, given that muscular power is an important determinant of effective physical functioning (Reid and Fielding, 2012), the effect of workplace HIIT on muscular power remains an interesting avenue for future research.

Lastly, we observed a small reduction in HDL-cholesterol, post-intervention (−0.1 mmol.L^−1^; −0.2 to 0.04 mmol.L^−1^, *d* = −0.2). Although a previous meta-analysis suggests aerobic exercise can increase HDL-cholesterol (Lin et al., 2015), the effect of HIIT on HDL-cholesterol in healthy populations is unclear and requires further investigation (Martland et al., 2020). Furthermore, workplace HIIT trials to date have reported no significant changes in HDL-cholesterol post-intervention (Shepherd et al., 2015; Allison et al., 2017). As HDL-cholesterol has a protective effect on coronary heart disease mortality (Cooney et al., 2009), this finding may be concerning and should be explored in future investigations. It is acknowledged that the post-prandial response for both glucose and triglycerides can be affected by a range of factors such as the content and timing of the previous meal and previous physical activity (Lopez-Miranda et al., 2007). While fasting status was included as a co-variate in the analysis, it is acknowledged that it was not possible to acutely control and replicate all of the factors that may have affected non-fasting blood lipid and glucose concentrations, and therefore this could be considered a limitation of this data.

We highlight the fact that we report here the results of an exploratory pilot study. As emphasised by multiple authors (Lee et al., 2014; Cipriani et al., 2015; Li et al., 2017) formal null hypothesis testing and the interpreting findings base on *P*-values is not consistent with our study approach. Rather, we have reported control-adjusted mean changes and associated 95% confidence intervals and effect sizes to explore the sensitivity of the outcomes. Therefore, it is important to quantify more precisely the mean treatment effects on these outcomes in a future definitive RCT. This future trial should be adequately powered and we have informed this issue via the reporting of change variance in predicted VO_2max_. Our findings also indicate that some of these outcomes can be feasibly measured in the workplace.

### Acceptability

The post-intervention focus groups provided a unique insight into participant perspectives of the acceptability of BE@Work. Here, participants generally reported that intervention logistics (e.g., frequency, length and location of HIIT sessions, exercise modes, group-based nature of the intervention and exercise facilitator) were satisfactory. This may be unsurprising as focus group participants attended an average of 21 out of 24 possible HIIT sessions. High session attendance could indicate that the participants were more likely to be satisfied with the intervention, as highlighted in a previous workplace HIIT intervention (Kinnafick et al., 2018), or it could be evidence of sampling bias. Regardless of views on intervention logistics, our participants considered flexibility in session attendance and exercise mode selection as instrumental in facilitating session attendance and enjoyment. While the scalability of providing a large volume of facilitated sessions across a week is questionable, computer assisted technology has shown promise for the delivery of workplace HIIT, thus eliminating the need for facilitated HIIT sessions (Metcalfe et al., 2020). While computer assisted HIIT may be regarded positively by some individuals, our participants reported that they enjoyed group-based facilitated HIIT, which highlights the need to cater to a wide range of exercise preferences in the workplace.

The BE@Work exercise modes (boxing, stair climbing and stair stepping) were selected by participants in focus groups conducted during intervention development (Burn et al., 2020). Post-intervention focus group participants regarded these exercise modes as acceptable and feasible, which highlights the importance of co-developing interventions with participants who are representative of potential study participants, in the settings in which interventions are to be delivered. Our participants highlighted the importance of having a choice in a variety of exercise modes within and between HIIT sessions, which has also been shown to enhance exercise adherence previously (Morgan et al., 2016). It is therefore surprising that most workplace HIIT interventions to date have used single exercise modalities throughout the intervention such as cycle ergometers (Shepherd et al., 2015; Metcalfe et al., 2020) or stair climbing (Allison et al., 2017). Our findings, along with recent work (Eather et al., 2020) highlight the utility of providing multi-modal HIIT in a workplace setting, which can cater to a wider range of exercise preferences.

When asked about their experiences during HIIT sessions, participants reported breathlessness, increased heart rate, local muscular fatigue and sweating; which were generally viewed as unpleasant or tiring. Previous laboratory-based work has highlighted that high-intensity exercise can result in unpleasant affective responses (Ekkekakis, 2003), which may in turn affect future exercise behaviour (Rhodes and Kates, 2015). Nonetheless, our participants also reported feeling alert during HIIT and energised afterwards. This positive post-exercise experience has been reported in previous laboratory-based work (Stork et al., 2020). In addition, social support and a positive group experience where individuals experience the same challenging situation has been reported to contribute to adherence in exercise interventions (Harden et al., 2015) and workplace HIIT interventions (Kinnafick et al., 2018). As our participants reported that the group-based nature of the intervention facilitated camaraderie and relationships with colleagues, it is possible that the group-based nature of the intervention engendered positive exercise experiences.

In line with previous work in laboratory-based (Stork et al., 2020) and fitness centre HIIT trials (Burn and Niven, 2018) our participants regarded the rest breaks incorporated into the HIIT protocol prescribed during BE@Work as particularly important. Participants reported that rest breaks made the intensity of HIIT intervals achievable and maintainable throughout the session. In addition to rest breaks, the HIIT prescribed in BE@Work was relative in nature, meaning that the intensity was individually prescribed to each participant based on their individual maximal heart rate. The work required to achieve a given percentage (e.g., >85%) of this heart rate depends on the fitness level of the participant, meaning that HIIT may be enacted differently in each person. While critics have postulated that HIIT will be perceived as too difficult, leading to poor adoption of the activity (Hardcastle et al., 2014; Biddle and Batterham, 2015), our participants highlighted that because they were not required to maintain pace with another participant or instructor, they could self-monitor the exercise intensity which made HIIT manageable and achievable.

Post-intervention, we observed evidence of improvements in markers of physical fitness (e.g., VO_2max_) and mental well-being (e.g., HR-QoL, perceived stress). Our focus group participants supported this finding, by reporting that they felt fitter and less stressed following the intervention. Given that participants of a previous workplace HIIT trial have also reported perceived changes in psycho-social outcomes post-intervention (Metcalfe et al., 2020), this suggests that a future trial could be designed to explore the effect of workplace HIIT with a psycho-social or mental health related primary outcome.

### Limitations

Although our study has produced some promising findings, it is not without limitations. Most notably, this was a pilot trial with exercise sessions delivered in a small sample of employees from one workplace. As such, the findings should be interpreted with caution. Despite high session attendance and promising preliminary implementation data, a range of generalisability biases may need to be considered ahead of a fully powered trial (Beets et al., 2020). Mostly notably, the scalability of delivering 19 HIIT sessions each week is clearly questionable and may lead to implementation support bias (Beets et al., 2020). The need for facilitated HIIT sessions could be overcome by the use of technology-based interventions as previously described (Metcalfe et al., 2020). Similarly, given that BE@Work was facilitated by a researcher experienced in the delivery of group-based exercise, the intervention may have been subject to intervention delivery agent bias. Future work could seek to train employees to deliver HIIT sessions, such as workplace wellness champions involved in previous workplace health promotion interventions (Ellis et al., 2021). Furthermore, participants in this study were healthy middle-aged adults working in office-based roles; therefore the findings cannot be extended to more diverse populations or workplaces. This could lead to target audience and setting bias (Beets et al., 2020). Critiques of HIIT commonly suggest that it will have limited appeal in unfit or inactive populations (Biddle and Batterham, 2015). Based on the baseline fitness of participants in the present study (VO_2max_ 33.5 mL·kg^−1^·min^−1^ in women and 42.7 mL·kg^−1^·min^−1^ in men), the effectiveness of HIIT in very deconditioned individuals cannot be addressed from the results of this study *per se*. However, the relatively large standard deviation of the baseline VO_2max_ (7.5 mL·kg^−1^·min^−1^) indicates that participants with a range of fitness levels were recruited to this study. Future studies could seek to implement workplace HIIT in a diverse range of workplace settings (e.g., manual labour settings) with a wider range of participants.

Randomisation to trial arms was not pragmatically possible in this study, because participating organisations were unable to accommodate exercise sessions at short notice. While this may have resulted in selection bias, allocation to groups was based on place of work rather than participant selection and baseline outcome values were included as a covariate in the statistical analysis, which may have partly mitigated this threat to validity. Another potential limitation relates to the use of a submaximal prediction of VO_2max_. While a gold standard VO_2max_ assessment was not logistically possible as previously described, it is acknowledged that the validity of predictive VO_2max_ assessments have been questioned (Grant et al., 1999). The use of non-fasted finger prick blood samples could also be considered a limitation. Although non-fasted blood samples appear to better predict cardiovascular disease risk (Nordestgaard et al., 2016) it is acknowledged that the post-prandial response for both glucose and triglycerides can be affected by the content and timing of the previous meal (Lopez-Miranda et al., 2007). Although, efforts were made to ensure the content and timing of meals were the same at both data collection time-points, and fasting status was included as a co-variate in the analysis, it is acknowledged that it was not possible to acutely control and replicate all of the factors that may have affected non-fasting blood lipid and glucose concentrations.

Upon trial registration, we had intended to assess acute mood and enjoyment responses in the intervention participants at fortnightly intervals. However, due to difficulties in obtaining responses from participants to acute data collection attempts, we could not robustly assess acute psychological responses to workplace HIIT, and therefore we have elected not to present this data. Nevertheless, this remains an interesting avenue for future investigation and should be considered when designing workplace HIIT trials.

## Conclusion

The BE@Work intervention was the mixed-methods pilot trial of a multi-activity workplace HIIT intervention. Low levels of drop-out, high session attendance, promising preliminary implementation data and generally positive participant perceptions indicate that multi-activity HIIT could be feasibly integrated into a workplace exercise intervention. While acknowledging the pilot nature of this data, compared with the control group, we observed preliminary evidence of improvements in predicted VO_2max_ post-intervention. Of the other outcomes, we observed evidence of changes in favour of the intervention group in two domains of HR-QoL (vitality and perceptions of general health), perceived stress, well-being and leg extensor muscle power. However, there was evidence of changes in favour of the controls for perceived pain, physical activity and HDL-cholesterol, which require further investigation. Our findings suggest that BE@Work could present an acceptable and viable workplace exercise intervention that may be engaging for some individuals. The findings of this pilot trial support the implementation of a definitive RCT exploring the effectiveness of workplace HIIT on physical fitness, cardiometabolic health and mental well-being.

## Data Availability Statement

The original contributions presented in the study are included in the article/Supplementary Material, further inquiries can be directed to the corresponding author.

## Ethics Statement

The studies involving human participants were reviewed and approved by Teesside University School of Health and Social Care Research Governance and Ethics Sub-committee (study number: 036/18). The patients/participants provided their written informed consent to participate in this study.

## Author Contributions

NB, MW, GA, and KW contributed to conception and design of the study. NB and KW contributed to the acquisition of data. NB, MW, GA, MG, and KW contributed to the analysis and interpretation of data. NB wrote the first draft of the manuscript. MW, GA, MG, and KW edited and wrote sections of the manuscript. All authors contributed to manuscript revision, read, and approved the submitted version.

## Conflict of Interest

The authors declare that the research was conducted in the absence of any commercial or financial relationships that could be construed as a potential conflict of interest.
